# Impact of comorbid conditions on asthmatic adults and children

**DOI:** 10.1038/s41533-020-00194-9

**Published:** 2020-08-20

**Authors:** Alan Kaplan, Stanley J. Szefler, David M. G. Halpin

**Affiliations:** 1 Family Physician Airways Group of Canada, 7335 Yonge Street, Thornhill, ON L3T2B2 Canada; 2 The Breathing Institute, Children’s Hospital of Colorado, 13123 E 16th Avenue, Box 395, Aurora, CO 80045 USA; 3 Royal Devon and Exeter Hospital, Barrack Road, Exeter, EX2 5DW UK

**Keywords:** Asthma, Asthma, Health care, Respiratory signs and symptoms

## Abstract

Comorbid conditions (comorbidities) can complicate the diagnosis and management of asthma. In different age groups, comorbid conditions can present varying challenges, including diagnostic confusion due to mimicking asthma symptoms, exacerbation of asthma symptoms, therapy for comorbid conditions affecting asthma or therapy for asthma affecting these conditions. This review aims to summarise some common comorbid conditions with asthma, such as rhinitis, vocal cord dysfunction, gastro-oesophageal reflux, psychiatric disorders, obesity and obstructive sleep apnoea, and discuss their prevalence, symptoms, diagnosis and treatment, highlighting any differences in how they impact children and adults. Overall, there is a lack of data on the impact of treating comorbid conditions on asthma outcomes and further studies are needed to guide age-appropriate asthma management in the presence of these conditions.

## Introduction

Comorbid conditions (comorbidities) in asthma—such as rhinitis, vocal cord dysfunction (VCD), gastro-oesophageal reflux disease (GERD), psychiatric disorders, obesity and obstructive sleep apnoea (OSA)—are common and often overlooked, leading to clinical confusion and complication of diagnosis^1^. Despite widespread occurrence, there does not appear to be a consensus on the definition of comorbid conditions in the literature^2^. They are sometimes defined as coexisting conditions and sometimes as conditions with causal connection, or in some instances, both^2–4^. In this review, we define comorbid conditions as disorders or diseases that are frequently present in a patient in addition to asthma. The comorbid conditions discussed may or may not have aetiological association with asthma, but their presence may nonetheless cause challenges in asthma management.

For patients with asthma, comorbid conditions carry a major economic burden. In the United States, 54% of adults with asthma in a nationally representative sample reported having ≥1 non-respiratory comorbid condition^5^. In a similar study in the UK on the prevalence of physical and mental health comorbid conditions, 63% of adults with asthma had ≥1 comorbid condition^6^. Furthermore, the number and prevalence of asthma comorbid conditions increases with age^7^, which is of particular concern given the globally ageing population^8^. Asthma comorbid conditions are associated with substantial healthcare costs that are five times higher than costs attributable to asthma alone^9^. On top of the direct costs, asthma comorbid conditions are associated with worse asthma-related outcomes^5,10^, increased risk of work disability^11^ and significant productivity losses^12^, further contributing to the burden of the disease.

Comorbid conditions can complicate the diagnosis and management of patients with asthma. Their symptoms may be similar to those associated with poor asthma control, which can lead to misdiagnosis and undertreatment or overtreatment. For example, obesity and asthma both cause breathlessness, putting patients at risk of a misdiagnosis. In fact, in one study, 36% of obese patients with physician-diagnosed asthma had no bronchial hyperresponsiveness, indicating a possible misdiagnosis^13^. As with obesity, comorbid conditions can also have a direct impact on respiratory symptoms, and their treatment can give rise to potential adverse events (AEs) associated with therapy^6,14^. For example, β-blockers for the management of cardiovascular disease, ocular hypertension or anxiety are avoided in asthma due to concerns over acute bronchoconstriction^6,15,16^.

Comorbid conditions are likely to lead to polypharmacy^14^, which in turn may have a negative impact on treatment adherence and asthma control^17^. Some asthma treatments may also increase the risk of developing comorbid conditions: inhaled corticosteroids (ICSs) in high doses can lead to osteoporosis^18^ and increase the risk of developing diabetes and pulmonary non-tuberculous mycobacterial infection^19,20^. There is therefore a potentially complex interplay between treatments and comorbidities; decision-making must take into consideration and balance potential benefits of treatment on one condition with the potential disadvantages on a comorbid condition.

Over the life course of patients with asthma, the prevalence and type of comorbid conditions may vary, further complicating asthma diagnosis and management. It is essential that clinicians treating patients with asthma understand all the comorbid conditions that are associated with asthma and identify their impact on different age groups. This will enable physicians and healthcare providers to recognise and manage asthma comorbid conditions in the most efficient and effective way. In this review, using literature searches on PubMed to identify review articles and manuscripts (initial search terms included ‘asthma[Title] AND comorbid*[Title]’, which were expanded as needed to identify literature), the authors critically review the literature and use their clinical knowledge and understanding to identify the comorbidities that are most frequently reported. During the review’s development, the authors held meetings to discuss and reach a consensus on which comorbidities were common and which would be discussed within the review. The authors acknowledge that most literature sources are from high-income countries (HICs), and therefore certain comorbidities that are more frequent in low- and middle-income countries (LMIC), for example, tuberculosis (TB), may not be discussed in this review. We define common comorbidities as those that are most frequently reported in literature and clinical practice from HICs.

**Fig. 1:**
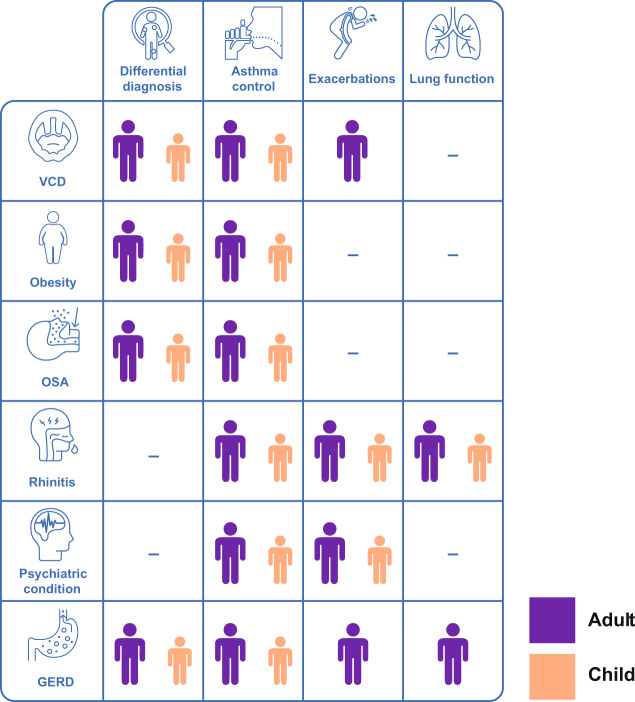
Interplay between comorbid conditions and aspects of asthma in adults and children. The comorbidities described in the figure, if present in the patient, may complicate the diagnosis of asthma. When seeking to achieve effective asthma management, it is important to address comorbidities. GERD gastro-oesophageal reflux disease, OSA obstructive sleep apnoea, VCD vocal cord dysfunction.

The overall aim of this review is to explore common comorbid conditions of asthma and highlight any differences in how they impact children and adults. The common comorbid conditions discussed in the review are summarised in Table 1. The complex interplay between comorbid conditions and aspects of asthma in adults and children is highlighted in Fig. 1. It emphasises that there may be comorbidities that need to be addressed in parallel to aspects of asthma in order to achieve effective asthma management, there may be comorbidities that complicate the diagnosis of asthma and the relative importance of these factors varies in adults and children.

**Table 1:** Prevalence, symptoms, diagnosis and associated asthma phenotypes of common comorbid conditions in children and adults with asthma.

Comorbid condition	Prevalence in children with asthma (%)	Prevalence in adults with asthma (%)	Symptoms	Diagnosis	Associated asthma phenotypes
(1) Rhinitis	59–78^1,2^	82–90^3,4^	• Nasal itching, sneezing, increased nasal secretions and nasal obstruction^5^ • General symptoms such as lassitude, cough and sleepiness may also occur^5^ as a consequence of sleep disturbances	• History and physical examination • Further testing for allergen-specific IgE antibodies and allergen skin prick testing • Validated questionnaires, such as Total Nasal Symptom Score and Sinonasal Questionnaire^6,7^	• Most commonly associated with early-onset allergic asthma phenotype^8^
(2) VCD	Not known	19–50^9,10^	• Chest tightness, wheezing (may be high pitched and musical or stridulous), hoarseness, dysphonia, cough and globus pharyngeus^11^	• Endoscopic examination^12^ • Spirometry^13^ • Pittsburgh VCD Index^13^	• Not associated with an asthma phenotype^8^
(3) GERD	43–87^14^	58 (includes patients aged 15–75 years)^15^	• Asthma symptoms worsen after consuming certain foods^16^ • Cough or wheezing after consuming acidic drinks or food or after a large meal^11,16^ • Hoarseness, predominance of nocturnal symptoms, symptom (heartburn, regurgitation) occurrence when changing position, unexplained dental decay^11,16^	• Trialling PPI^8^ • Specific examinations assessing GERD, such as impedance–pH monitoring and/or gastro-oesophageal endoscopy^12^	• Not associated with any asthma phenotype^8^
(4) Psychiatric diseases	Anxiety or depressive disorders: 16^17^	Any anxiety disorder: 34 (panic attacks, 25%; panic disorder, 12%; agoraphobia, 12%; generalised anxiety disorder, 9%)^18^	• Varies according to individual conditions	• Hospital Anxiety and Depression Scale questionnaire and psychiatric assessment^19^ • Patient Health Questionnaire (PHQ-9) for depression^20^ • Generalised Anxiety Disorder Questionnaire (GAD-7) for anxiety^21^	• Not associated with an asthma phenotype^8^
(5) Obesity	8–16^22^	21–48 (in severe asthma)^23–26^	• BMI ≥ 30 kg/m^2^^27^	• Measure weight and height to determine body mass index^27^	• At least two distinct phenotypes of asthma in obesity. Obese state can both alter early-onset allergic asthma and lead to the development of late-onset asthma^28^
(6) OSA	35–66^29,30^	40–50^31,32^	• Brief paroxysmal nocturnal dyspnoea, choking during sleep and poor sleep quality • Daytime sleepiness • Depression and memory loss^13,19,33^	• Polysomnography (gold standard)^19^ • Validated questionnaires, such as the Epworth Sleepiness Score, STOP-BANG or the Berlin Questionnaire are also available for screening^19^	• Not associated with an asthma phenotype^8^

## Rhinitis

### Prevalence

The prevalence of rhinitis in patients with asthma ranges from 6 to 95%, with the variability attributed to lack of standardisation in establishing a rhinitis diagnosis^21^. The literature suggests that both children and adults with asthma and comorbid rhinitis have more frequent physician visits, emergency room visits and hospital admissions and higher asthma-related drug expenses^22^. Upper airway pathologies such as nasal polyps often accompany asthma^23^. The presence of nasal polyps in asthma patients is associated with a more severe asthma phenotype as well as aspirin intolerance^23^ and may affect how asthma and other comorbid conditions are treated.

### Symptoms and diagnosis

Nasal itching, sneezing, increased nasal secretions, nasal obstruction and cough are common symptoms in patients with allergic rhinitis. However, rhinitis may also cause less obvious symptoms, such as lassitude and sleepiness, as a consequence of sleep disturbances^3,24^. If these are the presenting symptoms, the diagnosis of allergic rhinitis may be missed if the patient or caregiver is not actively questioned about nasal symptoms^3^. In a study in the USA, 53% of children with asthma and comorbid allergic rhinitis remained undiagnosed until they were clinically evaluated for the study^25^. Similar rates of underdiagnosis (32–45%) have also been reported in adults^26^ and could be due to under-reporting by patients or caregivers and poor recognition of rhinitis symptoms by physicians^27^.

As rhinitis and asthma tend to coexist, the Allergic Rhinitis and its Impact on Asthma guidelines highlight the importance of routinely investigating the presence of asthma in patients with persistent rhinitis^28^. Similarly, the Global Initiative for Asthma’s (GINA’s) Global Strategy for Asthma Management and Prevention and the National Asthma Education and Prevention Program’s Expert Panel Report for the diagnosis and management of asthma recommend an evaluation for comorbid allergic rhinitis in patients with asthma, especially in severe asthma^26,29^. Evaluation entails enquiring into patient history and making a physical examination. Patient history should include careful attention to environmental exposures, with a focus on precipitating factors and the impact of symptoms on quality of life^26^. Validated questionnaires such as the Total Nasal Symptom Score and Sinonasal Questionnaire may be used to give an objective assesssment^1,30,31^. Physical examination may show rhinorrhoea, enlargement and pallor of the inferior nasal turbinates, conjunctival infection and increased lacrimation (which are usually features of co-existent acute conjunctivitis), Dennie–Morgan lines (a fold in the skin below the lower eyelid), allergic shiners (dark circles under the eyes resembling bruises), a transverse nasal crease, sinus tenderness and nasal polyps^23,26^. Sensitisation to suspected allergens can be assessed by testing for allergen-specific immunoglobulin E (IgE) antibodies or allergen skin prick testing^26^. In vitro assays for serum allergen-specific IgE can be performed for patients who cannot have skin testing due to dermatographism or recent oral antihistamine use^26^. However, results of such allergen testing can be difficult to interpret in patients with asthma and may simply reflect exposure rather than causation of the symptoms.

### Impact of treatment on asthma outcomes

There is conflicting evidence on the benefits of adequately treating rhinitis in terms of its impact on asthma outcomes. One meta-analysis reported no significant improvement in asthma symptoms or lung function by treating rhinitis with intranasal corticosteroids, even though a trend in improvement was noted^32^. Other studies have shown that treatment does improve disease control and quality of life in asthma patients^22^. For example, in adults, the use of nasal corticosteroids was associated with a significantly reduced risk of asthma-related emergency room treatments and hospitalisations (adjusted odds ratio 0.75 [95% confidence interval (CI) 0.62–0.91] and 0.56 [95% CI 0.42–0.76], respectively)^3^. However, no similar studies have been reported in children^3^.

### Additional considerations

Consideration should be given to optimal management of sleep disturbances including sleep apnoea (a common comorbid condition in asthma) as a consequence of upper airway obstruction in patients with persistent asthma and comorbid rhinitis^4^. Sleep disturbances can lead to tiredness, irritability, memory deficit, daytime sleepiness and depression, thereby reducing quality of life^24^.

### Top tips for identifying rhinitis in primary care

- Patients may accept rhinitis as their ‘normal’, yet it can affect quality of life. Nasal congestion, rhinorrhoea and smell restrictions may be common, identifiable symptoms.

## Vocal cord dysfunction

### Prevalence

The prevalence of VCD is difficult to determine as it is a dynamic, episodic condition that may not be easily provoked at the time of examination^4^. In a study on patients with difficult-to-treat asthma, VCD was present in 50% of the patients as confirmed by computerised tomography^33^. Another study reported the prevalence of VCD, examined via laryngoscopy, to be 19% in patients with asthma^34^.

### Symptoms and diagnosis

The symptoms of VCD include chest tightness (which may be described as originating superior to the sternal notch), wheezing (may be high pitched and musical or stridulous), hoarseness, dysphonia, cough and globus pharyngeus^4^. These symptoms may be provoked by respiratory or laryngeal irritants, exercise stress, anxiety or even by frequent use of inhalers^4^. These symptoms also overlap with asthma symptoms, which can lead to both inaccurate diagnosis and incorrect assessment of the severity of the asthma^1^.

VCD is often suspected by the increase in the level of symptoms at the time of physical examination and by the presence of audible wheeze from the mouth over the lung fields on auscultation^35^. Diagnosis of VCD can be difficult to confirm as physical examination and spirometry may be normal between episodes^36^. It is also important to exclude significant upper airway pathology such as tracheobronchial tumours or subglottic stenoses before considering a diagnosis of VCD^37,38^. The gold standard for diagnosing VCD is endoscopic examination with direct visualisation of the vocal cords via laryngoscopy, possibly after bronchial challenge or during an acute attack^39^. To evaluate whether vocal cord movement is abnormal, patients should also be instructed to sniff, verbalise sounds through sequential phonation, breathe, pant and carry out repetitive deep breaths^40^. During spirometry, a truncated inspiratory loop may also help to diagnose VCD in the presence of symptoms but the predictive value for this process is low^36^. Questionnaires such as the Pittsburgh VCD Index have a good predictive value to diagnose VCD (cut-off score ≥4) and may be helpful to distinguish VCD from asthma^36^. Patients with poor asthma control, frequent exacerbations or inadequate response to treatment should be investigated for VCD^36^.

### Impact of treatment on asthma outcomes

Treating VCD involves a multidisciplinary approach. Speech therapy is considered the cornerstone of VCD treatment^1,36^. Other therapies such as continuous positive airway pressure (CPAP), injecting *Clostridium botulinum* toxin into laryngeal muscles or psychotherapy have been suggested as beneficial, but no convincing data exist to support their use^1,36^.

Identifying and successfully treating VCD can have a significant impact on asthma outcomes. A recent prospective observational study showed that diagnosis and treatment of VCD led to a decline in asthma medication use and improvement of symptoms in 79 and 82% of patients, respectively^41^. In another study, speech pathology treatment for VCD reduced asthma medication use in 80% of female adolescent athletes^42^.

### Additional considerations

The most important issues with VCD are that it is sometimes misdiagnosed as, and often complicates, asthma^1,4^. This can lead to excessive or inappropriate use of asthma medications^40^.

### Top tips for identifying VCD in primary care

- VCD can be difficult to diagnose but should be considered when patients have persistent wheeze at rest despite using inhaled bronchodilators.

## Gastro-oesophageal reflux (GERD)

### Prevalence

In a systematic review of studies based on questionnaires, personal interviews or databases, the prevalence of GERD (defined as at least weekly heartburn and/or acid regurgitation) in patients with asthma was 58%^43^. In studies where GERD was confirmed through oesophageal pH monitoring, the pooled sample-size-weighted average prevalence of abnormal oesophageal acid exposure in asthma patients was 51%^43^.

### Symptoms and diagnosis

In patients with asthma, clinicians should suspect comorbid GERD if asthma symptoms worsen after certain foods (e.g. chocolate, alcohol, peppermint, coffee, etc.) or patients experience cough or wheezing after acidic drinks or food or after a large meal^44^. Other symptoms that could indicate comorbid GERD include hoarseness, predominance of nocturnal symptoms, symptom (heartburn, regurgitation) occurrence when changing position and unexplained dental erosion^44,45^.

GERD can pose a diagnostic challenge as the disease may have atypical or extra-oesophageal symptoms, such as chronic cough, laryngitis and non-cardiac chest pain^45^. These atypical manifestations can be challenging to diagnose in the absence of the typical symptoms of heartburn and regurgitation^45^. Symptoms such as cough, laryngeal or pharyngeal irritation and chest tightness overlap with those seen in individuals with asthma and/or upper airway disease, making the identification of GERD a challenge, and potentially leading to inappropriate choice of therapy^4,46^.

Empiric therapy with twice-daily proton pump inhibitor (PPI) is the recommended management option in patients suspected of having GERD-related complications^1,47^. However, some evidence suggests that PPI therapy may provide little to no improvement in asthma control^48^. To further confirm diagnosis of GERD as a comorbid condition, specific examinations assessing for GERD, such as impedance–pH monitoring and/or gastro-oesophageal endoscopy, can be used^39^. The American Academy of Allergy Asthma and Immunology advise that lifestyle changes including weight loss, a reduction in alcohol consumption and smoking cessation can help with the management of GERD. In addition, elevating the head of the bed or advising the patient not to lie down within 2–3 h of eating may assist in reducing symptoms of GERD^49^.

### Impact of treatment on asthma outcomes

Improvements in lung function, symptoms and quality of life have been reported in a few studies of PPI treatment in patients with asthma and comorbid GERD^1,50–54^. However, other studies reported no measurable improvement in asthma control^48,55^. A Cochrane review by Chan et al. concluded that there is inadequate evidence to recommend empirical use of PPIs for routine treatment in asthma^56^. Even though evidence of the impact of treating GERD on asthma outcomes is somewhat conflicting, referral to a gastroenterologist should be considered if GERD is not controlled on twice-daily PPI and is required if the patient has symptoms such as dysphagia, odynophagia, involuntary weight loss or anaemia^1^.

### Additional considerations

Some asthma therapies may aggravate GERD and even, paradoxically, worsen asthma control^57^. For example, theophylline, albuterol and bronchodilators may decrease lower oesophageal sphincter tone leading to increased GERD, systemic corticosteroids may increase gastric acid production and ICS may lead to hoarseness similar to that caused by GERD^4^. GERD is also a frequent complication of pregnancy and is reported to be more severe in pregnant women with asthma than in pregnant women without asthma^58^.

### Top tips for identifying GERD in primary care

- While classic symptoms of heartburn and food and acid reflux may be present, they may also be absent. Therefore, it is important to look for any unexpected dental issues and changes in the patient’s voice.

## Psychiatric diseases

### Prevalence

Estimates of the prevalence of psychological disturbances among patients with asthma vary widely. A World Health Organization survey of psychiatric comorbid conditions in 85,000 patients with asthma, using a standardised, structured psychiatric interview with trained interviewers, reported an estimated prevalence of 2–26% for major depression^59^. In the same study, age-matched patients without asthma had an estimated prevalence of 1–9% for major depression^59^. Anxiety is also a commonly reported comorbidity in patients, with 1 Italian study in 263 outpatients with asthma reporting that a third of patients had anxiety. Furthermore, the authors reported that patients who report anxiety have a greater perception of worse asthma control than those with no anxiety^60^.

The prevalence of comorbid mental and behavioural disorders appears to increase with age. For example, in a study using routine healthcare data of German children and adolescents with asthma, the prevalence of depression increased from 0.3 to 3% and anxiety disorder increased from 2 to 5% between 6–9- and 14–17-year-olds^61^. In a Spanish National Health survey, factors such as older age and concomitant comorbid conditions were associated with higher rates of depression and anxiety in patients with asthma^62^.

### Symptoms and diagnosis

Patients with moderate-to-severe asthma or difficult-to-control asthma should be assessed for depression, panic and anxiety disorder. In order to confirm the diagnosis and follow-up response, it is advised to use a validated questionnaire such as the Hospital Anxiety and Depression Scale^1^, the Patient Health Questionnaire (PHQ-9) for depression and the Generalised Anxiety Disorder Questionnaire (GAD-7) for anxiety^63,64^.

Comorbid depressive disorders may commonly go unrecognised and untreated in children with asthma. Even if mental disorders are recognised, only one in five children with asthma receive adequate treatment^3^. In adolescents, risk behaviours are common and tend to co-occur with peer conflict, troubled parent–adolescent relationships and mood disorder. Depression, in particular, can lead to a sense of hopelessness that can negatively affect adherence^65^. Due to rapid physical, emotional, cognitive and social changes in adolescence, managing asthma and comorbid conditions in this group can be challenging^29^. Exploratory and risk-taking behaviours, such as smoking and inhaled substance use, occur at a higher rate in adolescents with chronic diseases than in healthy adolescents^29,66^. It is important to assess risk behaviours, such as smoking, alcohol and other substance abuse, in children and adolescents. During consultations, the GINA strategy recommends that adolescents should be seen separately from the parent/carer so that sensitive issues, such as smoking, adherence and mental health, can be discussed privately and confidentiality agreed^29^. In case of significant psychological symptoms, patients should be referred to local mental health services or resources, as available.

### Impact of treatment on asthma outcomes

Only a few studies are available investigating the impact of pharmacological treatment of comorbid psychiatric diseases on asthma control. In a 12-week randomised controlled trial in 90 adults with asthma and major depressive disorder, the effect of antidepressant medications on asthma control was studied versus placebo^67^. Although no difference in depression scores was observed between patients treated with antidepressants and those on placebo, use of oral corticosteroids was lower in antidepressant-treated patients and a reduction in depressive symptoms was associated with an improvement in asthma^67^. Another similar, but more recent, trial reported a significant reduction in Asthma Control Questionnaire score and oral corticosteroid use in 21 patients with more severe asthma on antidepressant treatment compared with placebo^68^. Treating depressive symptoms may improve asthma outcomes, but more evidence is required.

### Additional considerations

Potential effects of asthma medications on mental health should also be considered. Adverse effects such as mood and behavioural changes, including manic or depressive states and even frank psychosis, can occur with large doses of oral corticosteroids^69^. Concerns about a possible association between leukotriene receptor antagonist use and suicide risk were raised based on post-marketing surveillance reports^69^. Even though a statistically significant association was found in patients aged 19–24 years in a case–control study, the association was no longer significant after adjusting for potential confounding factors^69^. In some children, post-marketing surveillance reports have identified behavioural and/or neuropsychiatric AEs associated with montelukast use^70^, and a possible association with suicide risk has been reported in adolescents and adults^71^. Since March 2020, the Food and Drug Administration has required a boxed warning about the risk of neuropsychiatric events with montelukast to strengthen an existing warning^72^. In addition, the potential effects of medications for psychiatric diseases on asthma control should be considered. For instance, β-blockers—a commonly prescribed treatment for anxiety—are contraindicated in asthma and may cause exacerbations. The benefit of a treatment for one condition should, therefore, be quantified against the potential risks of the comorbidity^73^.

More important is the potential impact of depression and anxiety on asthma management. For example, depression may affect medication adherence^74^, while anxiety associated with hyperventilation may result in misinterpretation of symptoms as asthma^75^.

### Top tips for identifying psychiatric diseases in primary care

- If asthma control is poor, patients should be assessed for unrecognised anxiety and depression, along with impact on day-to-day functioning.

## Obesity

### Prevalence

Obesity is a common comorbid condition in both children and adults with asthma, and it is present in 21–48% of patients with severe asthma^76–79^. In the general population, the prevalence of obesity varies by country and ranges from 4 to 38%^80^. In a study of 6–17-year-old patients with asthma, obesity appeared to increase with age (8% in 6–9-year-olds, 14% in 10–13-year-olds and 16% in 14–17-year-olds)^61^.

Obesity, in itself, is a major public health problem. The prevalence of obesity has more than quadrupled from 1971 to 2006 in children aged 6–11 years in the USA, as well as increasing sharply for adolescents^3,81^. In a more recent survey in 2015–2016 in the USA, the prevalence of obesity was recorded to be 40% in adults and 19% in adolescents, both exhibiting higher prevalence rates in 2016 compared with rates in 1999^82^.

Obesity can lead to the development or worsening of asthma via mechanical, inflammatory and genetic/developmental factors^83^. OSA and GERD in obese patients may also lead to worse asthma symptoms^83^.

Obesity may impact asthma differently in younger age groups compared with adults^62^. Studies have shown that the effect of obesity is different and more pronounced in early- versus late-onset asthma. Persistent asthma beginning in childhood may be significantly complicated by obesity developing later in life^62^. It has been suggested that late-onset asthma in an already obese patient may not be complicated further by obesity, as obesity may have already been a major factor in its appearance^62^. However, results from the Severe Asthma Research Program have shown that obese late-onset asthmatics are more likely to have been admitted to the emergency department or intensive care unit versus non-obese late-onset asthmatics^84^, suggesting their symptoms may be accentuated and management more complicated as a result of their disease phenotype. Therefore, it is important to differentiate between patients having asthma and becoming obese later and obese patients developing new-onset asthma^62^.

### Symptoms and diagnosis

Obesity is defined as having a body mass index (BMI) ≥ 30 kg/m^2^^69^. Obese patients can present with respiratory symptoms such as breathlessness on exertion, which may be mistaken for asthma. Therefore, before making a diagnosis of asthma it is important to demonstrate the presence of variable airflow limitation^69^.

### Impact of treatment on asthma outcomes

A recent systematic review of 10 randomised controlled trials explored the effect of weight loss on asthma outcomes in obese children and adults^85^. Weight loss interventions ranged from dietary restrictions to multifactorial interventions with exercise training and cognitive behavioural therapy, with a duration of 8 weeks to 18 months^85^. Most of the studies reported improvements in asthma-related quality of life and, to some degree, asthma control, and the systematic review concluded that weight loss in asthma patients with comorbid obesity may improve asthma outcomes^85^. Evidence for an association between bariatric surgery and improvements in asthma outcomes—such as improved Asthma Control Test (ACT) scores, asthma control and quality of life—have also been reported in some studies^86^.

### Additional considerations

Obese patients with asthma may have a reduced response to asthma treatments, such as ICS, compared with non-obese patients^87^. A specific phenotype of asthma exists in some obese patients. This is associated with lung function changes due to breathing at low lung volumes, a systemic inflammatory process that may possibly influence airways and a reduced response to asthma medications^22^. Asthma and obesity can both influence the onset of GERD and sleep disturbances, which can mimic the ‘obese asthma’ phenotype and lead to misdiagnosis^88^.

### Top tips for identifying obesity in primary care

- There is value in measuring and documenting height, weight and BMI.

## Obstructive sleep apnoea

### Prevalence

The prevalence of OSA in children with asthma ranges from 35 to 66%^89,90^; in adults, prevalence is reported to be 40–50%^91,92^. In the general population, estimates of OSA prevalence are in the range of 3–7%^93^. OSA is associated with more severe exacerbations in adults and poorer asthma control in children^36^. The causes of developing OSA across age groups are generally different; in children, enlarged adenoids and tonsils and nasal obstruction have been identified as primary reasons, whereas in adults, a mixture of aetiological factors such as anatomical variations, age, male sex, ethnic difference and obesity may lead to increased collapse potential of the pharyngeal muscles^36^. Smoking, diabetes, hypothyroidism, alcohol consumption and medication usage are all factors that could contribute to OSA^36^. Two mechanisms have been proposed for how OSA may impact asthma control: it may increase neutrophilic airway inflammation or lead to vagal activation from the collapsed pharynx leading to increased bronchial hyperresponsiveness^94^.

### Symptoms and diagnosis

OSA is characterised by total or partial repetitive obstruction of the upper airway during sleep, leading to poor quality of sleep, with symptoms such as brief paroxysmal nocturnal dyspnoea, choking during sleep, daytime sleepiness, depression and memory loss^36,94,95^. OSA may also aggravate or mimic asthma symptoms^36,94^, and both asthma and OSA are associated with airway obstruction and have many diurnal and nocturnal symptoms in common^95^.

Polysomnography is the gold standard for diagnosing OSA^94^. However, if polysomnography is not available, overnight pulse oximetry can also be used as a screening tool^96^. Validated questionnaires such as the Epworth Sleepiness Score, STOP-Bang or the Berlin Questionnaire are also available for screening of OSA^94^. In particular, special attention should be given to screening OSA in children with asthma and coexisting allergic rhinitis, as well as adenotonsillar hypertrophy^36,97^. In the severe asthma population, patients with OSA may not have classic symptoms of sleep apnoea and diagnosis may be overlooked^94^.

### Impact of treatment on asthma outcomes

CPAP is the first line of treatment for OSA and its use can have a positive impact on asthma outcomes^98^. In a survey-based study where asthmatic patients with OSA initiated CPAP therapy after starting asthma medication, there was a significant reduction in self-reported asthma severity and the number of patients using rescue medication and an increase in ACT score^99^. Larger multi-country observational studies are required to fully evaluate the impact of CPAP on asthma outcomes.

### Additional considerations

Poorly controlled OSA adversely affects sleep, mood and lung function, so treatment is paramount^36,94,95^.

### Top tips for identifying OSA in primary care

- Patients who are identified to snore or who have unrestorative sleep with other comorbidities, such as diabetes, cardiovascular disease and obesity, are at high risk of OSA^100^. Therefore, the STOP-Bang screening tool^101^ or Epworth Sleepiness Score (used as a scale to measure sedation)^102^ are useful tools for OSA.

## Other comorbid conditions in children

Other common comorbid conditions in children with asthma include chronic sinusitis and respiratory infections^103^. Infants with food allergies have approximately double the chance of developing asthma before they reach school age^104^, and comorbid food allergy is a significant risk factor for life-threatening asthma; in a small study, 52.6% (*n* = 10) of severe asthma cases had food allergy compared with only 10.5% (*n* = 4) of the controls (*P* = 0.006)^105^. Regular assessments are required in these patients, including dietary and emergency management plans, and treatment adherence reviews^106^.

Top tips for identifying other comorbid conditions in children in primary care

- The effect of comorbidities in paediatric patients with asthma who have a history of hives, anaphylaxis and food intolerances should be considered.

## Other comorbid conditions in adults

Multimorbidity is common in adults with asthma and the number of comorbid conditions in asthma increases as patients age^62,107^. The types of comorbid conditions differ by age; for example, in a sample population of >1 million patients with asthma, 47% of adults aged ≥55 years suffered from comorbid hypertension compared with 12% in 18–55-year-olds and 0.1% aged <18 years^108^. The prevalence of comorbid diabetes, congestive heart failure and fluid and electrolyte disorders was also significantly higher in the ≥55-year age group, followed by 18–55 years and then <18 years^108^. Another study identified hypertension (20.1%), pain (15.9%), chronic obstructive pulmonary disease (COPD; 13.4%) and dyspepsia (10.9%) as the most frequent comorbid conditions in adults with asthma^6^. COPD, in particular, usually affects people aged ≥40 years and can coexist with asthma^62,108^. The GINA strategy and the Global Initiative for Chronic Obstructive Lung Disease provide recommendations and treatment guidelines for patients with asthma–COPD overlap^29,62^. Some patients with asthma also have chronic pain, which may be treated with opiates; however, while hypersensitivity to, or accumulation of, opiates is associated with respiratory depression, it is a very rare phenomenon^109^.

Top tips for identifying other comorbid conditions in adults in primary care

- It is important to perform a complete history and physical examination to ensure that any comorbid conditions the patient may have are considered and evaluated.

## Discussion

The presence of comorbid conditions complicates the management of asthma in all age groups. As clinical trials generally exclude elderly patients or asthma patients with serious comorbid conditions, there is a lack of strong evidence to guide asthma treatments in these individuals^62,69^. Many comorbid conditions may impact asthma directly and some may require medications that can worsen asthma, such as bronchoconstriction-related AEs from β-blockers and non-steroidal anti-inflammatory drugs (NSAIDs)^16,110^. The presence of diabetes can affect decisions about the use of systemic corticosteroids, which are known to cause metabolic complications in glucose homoeostasis, including insulin resistance and hyperglycaemia^20,69^. In patients with osteoporosis, vertebral fractures can impair respiratory capacity^69^. In addition, other common age-related problems such as osteoarthritis, cognitive impairment, poor eyesight, hearing loss or poor coordination can hamper a patient’s ability to use their inhaler device correctly^69^.

As previously discussed, it should be acknowledged that a potential limitation of this review is that it focuses on comorbidities most frequently reported in HICs. Therefore, comorbidities affecting patients with asthma from LMICs may vary and should be addressed accordingly. For example, in countries with a high TB burden, treatments for patients with asthma should be considered carefully as there may be an increased risk of latent TB reactivation following oral corticosteroids or ICS use^111,112^. Furthermore, symptoms such as chronic cough may relate to asthma or TB; therefore, if the presence of TB has been excluded, asthma should be investigated^113^. In addition to geographical influencing factors, factors such as medication side effects and age can influence comorbidities and asthma control and should be considered during clinical decision-making.

In conclusion, comorbid conditions can complicate asthma management in multiple ways; they may be part of the same pathophysiological process as asthma (e.g. rhinitis) or may mimic and/or exacerbate asthma symptoms (e.g. GERD and VCD). In some cases, comorbid conditions may affect asthma therapy (e.g. diabetes) or therapy for comorbid conditions may affect asthma (e.g. β-blockers and NSAIDs). Understanding the pattern of comorbid conditions across the life course of asthma patients is important as this will help healthcare professionals to make accurate diagnoses, facilitate prescription of appropriate therapy and improve asthma management. Further studies evaluating changes in comorbid conditions over time and their impact on asthma control are needed to provide clear age-related guidance on managing asthma patients with comorbid conditions.

### Reporting summary

Further information on experimental design is available in the Nature Research Reporting Summary linked to this paper.

## Supplementary information

Reporting summary
